# Development of a Chimeric Vaccine Providing Protection Against the Type A ASIA/Sea-97 FMDVs in East Asia

**DOI:** 10.3390/vaccines13111104

**Published:** 2025-10-29

**Authors:** Sungho Shin, Seong Yun Hwang, Mi-Kyeong Ko, Min Ja Lee, Su-Mi Kim, Jaejo Kim, Jong-Hyeon Park

**Affiliations:** Animal and Plant Quarantine Agency, 177 Hyeoksin 8-ro, Gimcheon City 39660, Gyeongsangbuk-do, Republic of Korea; imshin121@korea.kr (S.S.); hsy8592@korea.kr (S.Y.H.); mkk80@korea.kr (M.-K.K.); herb12@korea.kr (M.J.L.); beliefsk@korea.kr (S.-M.K.); jkim1209@korea.kr (J.K.)

**Keywords:** foot-and-mouth disease (FMD), swine, chimeric vaccine, type A, East Asia

## Abstract

**Background/Objectives:** Foot-and-mouth disease (FMD) remains a significant threat to livestock, particularly in the pool 1 region (East Asia), where serotype A is prevalent. Vaccination is the most effective control measure, and the selection of the appropriate vaccine strain is critical for ensuring effective protection. The A/ASIA/Sea-97 lineage (and its G1 and G2 sublineages) has been reported in this region, necessitating the development of an appropriate vaccine. This study aimed to develop a potent candidate vaccine strain capable of providing effective protection against the G1 and G2 sublineages of the A/ASIA/Sea-97 lineage. **Methods:** Chimeric vaccine development was achieved by replacing and inserting antigenic sites derived from the A/ASIA/Sea-97 G1 (VP4, VP2, and VP3) and G2 sublineage (VP1 and GH loop) strains. The candidate strains were evaluated for protective efficacy in mice and pigs. **Results:** In mice, the two candidate vaccines provided strong protection against challenge with a G1 sublineage virus (A/POC/2010) and A22 Iraq and two G2 sublineage viruses (A/YC/2017 and A/GP/2018). Subsequently, the most effective candidate was selected for testing in pigs. One month after vaccination, the pigs were protected against two A/ASIA/Sea-97 viruses (A/POC/2010 and A/GP/2018) prevalent in East Asia. **Conclusions:** These results demonstrate that the developed strain has significant potential as a vaccine against the type A FMD viruses circulating in East Asia and that vaccination with this strain could be an effective strategy for regional FMD control.

## 1. Introduction

Foot-and-mouth disease (FMD) is a highly contagious viral disease of cloven-hoofed animals such as cattle and pigs, which is caused by the foot-and-mouth disease virus (FMDV). The infection induces vesicular lesions on the mouth and feet, spreads rapidly, and causes severe economic losses to the livestock industry [1].

Vaccination remains the most effective measure for FMD control [2]. Its success depends on selecting vaccine strains that antigenically match circulating viruses [3], which helps prevent virus spread and economic damage, highlighting its central role in FMD control [4].

Globally, seven serotypes of FMDV—O, A, C, SAT1, SAT2, SAT3, and Asia1—have been identified, with regional differences in prevalence. The SAT serotypes dominate in Africa, while serotypes O, A, and Asia1 are common in the Middle East. In Asia (pool 1 region), serotypes O, A, and Asia1 are most frequently reported [5,6]. These regional patterns are key considerations for vaccine development.

Among these, serotype A exhibits the greatest antigenic diversity, and cross-protection among its lineages is often incomplete, reducing vaccine efficacy [7,8]. This high variability is largely attributed to the low fidelity of the viral RNA polymerase, which leads to frequent mutations during replication [9]. Consequently, antigenic variants frequently emerge, and vaccine strains can become outdated within a few years, necessitating periodic updates every 5–10 years [10].

Outbreaks of the A/ASIA/Sea-97 lineage occurred across Southeast, Central, and East Asia between 2009 and 2020, including in the Republic of Korea, Mongolia, Laos, and Thailand. However, commercial vaccine strains often fail to match this lineage, leading to insufficient protection [11]. A recent phylogenetic study reported rapid genetic diversification and geographic spread within the lineage, underscoring the need for timely re-selection of vaccine strains [12].

In the Republic of Korea, three FMD outbreaks caused by serotype A viruses have been recorded since 2010. The 2010 outbreak was associated with the A/ASIA/Sea-97 G1 sublineage (A/POC/2010), while the 2017 and 2018 outbreaks were linked to G2 sublineage strains (A/YC/2017 and A/GP/2018) [13].

Unlike our previous study that pursued broad-spectrum protection across topotypes [14], the present study aimed to develop a vaccine optimized for the A/ASIA/Sea-97 lineage, targeting the G1 and G2 sublineages currently circulating in East Asia. This represents a region-specific vaccine strategy consistent with recent epidemiological trends.

This study’s objective was to develop a vaccine candidate with improved protection by incorporating the P1 structural-protein regions from the A/POC/2010 (G1) and A/GP/2018 (G2) strains. We anticipated that this candidate would confer broad protection against A/ASIA/Sea-97 viruses and contribute to effective FMD control in Asia.

## 2. Materials and Methods

### 2.1. Cells

Fetal goat tongue (ZZ-R 127; Friedrich-Loeffler-Institut, Riems, Germany) cells were propagated in Dulbecco’s modified Eagle’s medium (DMEM/F12; Corning, Union City, NJ, USA). Baby hamster kidney (BHK-21; C-13, ATCC CCL2-10, Manassas, VA, USA) and porcine kidney (LFBK; Plum Island Animal Disease Center, Orient, NY, USA) cells were cultured in DMEM supplemented with 10% fetal bovine serum (FBS; Gibco, Paisley, Renfrewshire, UK) and 1% antibiotic–antimycotic (Gibco, Paisley, Renfrewshire, UK) at 37 °C in a 5% CO_2_ humidified incubator. Suspension-adapted BHK-21 cells, developed by the Animal and Plant Quarantine Agency (APQA) and the Korea Research Institute of Bioscience and Biotechnology in the Republic of Korea, were grown in CD BHK-21 Production Medium (Gibco, Paisley, Renfrewshire, UK) containing sodium bicarbonate (Sigma-Aldrich, St. Louis, MO, USA) and maintained in a shaking CO_2_ incubator under the same conditions.

### 2.2. Viruses

The FMDV sequences used in this study were O_1_Manisa/Turkey/69 (O_1_Manisa, GenBank accession AY593823.1), A/Pocheon/001/KOR/2010 (A/POC/2010, GenBank accession KC588943.1), A22 Iraq/24/64 (A22 Iraq, GenBank accession AY593763.1), A/YC/SKR/2017 (A/YC/2017, GenBank accession KY766148.1), and A/SKR/4/2018 (A/GP/2018, GenBank accession MK463492.1).

### 2.3. Targeted Mutagenesis and Subcloning

The chimeric viruses were constructed as previously described [15]. A C142T mutation was introduced into the 3C protease by polymerase chain reaction (PCR) using the forward primers 5′-CCATGGATGGAGACACCATG-3′ and the reverse primer 5′-CACTACAATGTCTTTGTAGGTA-3′. The 3B1 and 3B2 regions were replaced with duplicated 3B3 sequences amplified from a cDNA vector using forward primer 5′-AGGACCGACCACAAGCTGAAGGACCTTACGAGGGACCGGT-3′ and reverse primer 5′-TCGGTCGGTGGGGCACCACTCTCAGTGACAATCAAGTTCT-3′. VP4, VP2, VP3, and VP1 of O_1_Manisa P1 were replaced by those of A/POC/2010 P1 (GenBank accession. KC588943.1), and VP1 was then replaced by VP1 of A22 Iraq (GenBank No. AY593763.1) to create the vaccine strain “Apo22” [14]. Another strain, “Apo22-GP”, was generated by inserting amino acids 140–160 of A/GP/2018 VP1 (GenBank No. MK463492.1) into the Apo22 VP1.

This procedure was performed with Phusion High-Fidelity DNA Polymerase (Thermo Fisher Scientific, Vantaa, Finland) in accordance with the supplier’s protocol, and the constructs were sequence-verified by Macrogen Corporation (Geumcheon-gu, Seoul, Republic of Korea). The primers used in the cloning were 5′-TGGTGAAACCCGCCACACAGCTCCCTGCCTCCGCGAGGGTCGCCGCTCAG-3′ (Apo22-GP forward) and 5′-CGAGAGGACCCGAGTCACCTCGCCGGTTTTGCGCGAGAGGCCCTAGGTC-3′ (Apo22-GP reverse).

### 2.4. Virus Recovery and Cell Culture

The virus recovery process followed a procedure in a previous study [16]. Recombinant cDNA plasmids were digested with the restriction enzyme SpeI (NEB, Ipswich, MA, USA) and then transfected into BHK/T7-9 cells, which express T7 RNA polymerase, using Lipofectamine 3000 (Invitrogen, Waltham, MA, USA). Following a 72 h incubation at 37 °C in a humidified 5% CO_2_ environment, virions were collected using three freeze–thaw cycles. The resulting chimeric viruses were then expanded, first in fresh ZZ-R 127 cells and, subsequently, in BHK-21 cells.

### 2.5. Viral Cultivation and Purification

The virus was propagated in suspension-adapted BHK-21 cells under orbital shaking for 16 h until cytopathic effect (CPE) appeared. For antigen production, the working virus was passaged six times on adherent ZZ-R cells, seven times on adherent BHK-21 cells, and five times in suspension-adapted BHK-21 cells. Virions were harvested by repeated freeze–thaw cycles followed by centrifugation (4000 rpm, 20 min, 4 °C) to remove debris. Inactivation was carried out with 0.003 N binary ethylenimine (BEI; Sigma-Aldrich, St. Louis, MO, USA) under gentle agitation (75 rpm) at 26 °C for 24 h, and residual BEI was neutralized with 10% 1 M sodium thiosulfate (final 2%; Daejung Chemicals, Siheung, Republic of Korea). Virus inactivation was confirmed by passaging ZZ-R 127 and BHK-21 cells twice in the presence of the solution containing the inactivated virions. The inactivated virus was precipitated overnight at 4 °C with 7.5% polyethylene glycol (PEG) 6000 and 2.3% sodium chloride (both from Sigma-Aldrich, St. Louis, MO, USA), resuspended in Tris-NaCl buffer, and purified by ultracentrifugation through a continuous 15–45% sucrose density gradient at 30,000 rpm (SW41 rotor) for 4 h at 4 °C. Antigen concentration was measured spectrophotometrically at 259 nm, and virion integrity was assessed using a transmission electron microscope (Hitachi H7100FA, Tokyo, Japan).

### 2.6. Preparation of the Candidate Vaccines

The candidate vaccines were prepared as previously described [14]. Each vaccine was prepared using 15 μg of purified Apo22 and Apo22-GP 146S antigens mixed 1:1 (*v*/*v*) with ISA 206VG (Seppic, Paris, France). Subsequently, 10% aluminum hydroxide gel (Rehyragel HPA, General Chemical, Moorestown, NJ, USA) and 0.5 μg of saponin (Sigma-Aldrich, St. Louis, MO, USA) were incorporated in sequence to generate a water-in-oil-in-water (W/O/W) emulsion. The vaccine was prepared one day before vaccination. Vaccine formulations intended for mouse immunization were produced using the same procedure but with proportionally lower antigen doses. All vaccines were formulated one day prior to vaccination, stored at 4 °C until use, and gently mixed immediately before vaccination.

### 2.7. Vaccination and Virus Challenge in Mice

Female C57BL/6 mice, aged seven weeks (KOSA-BIO, Seongnam, Republic of Korea), were vaccinated with Apo22 or Apo22-GP at doses equivalent to 0, 1/10, 1/40, 1/160, and 1/640 of the dose used in the pigs (15 μg of antigen). Optional doses of 1/1280 and 1/2560 were also tested in some cases. At seven days post-vaccination (dpv), mice were challenged intraperitoneally with 1 × 10^5^ TCID_50_ per 0.1 mL of virus and monitored for seven days. The 50% protective dose (PD_50_) was calculated using the Spearman–Kärber method, as recommended by the World Organization for Animal Health [17]. Vaccine dilutions were based on antigen quantity (μg), and 3 to 5 mice were used per group depending on the design (Supplementary Table S1).

### 2.8. Vaccination and Virus Challenge in Pigs

Eight pigs received a single intramuscular dose of the Apo22-GP vaccine (15 μg/mL of antigen). At 28 dpv, animals were challenged via the footpad with either A/POC/2010 (*n* = 6) or A/GP/2018 (*n* = 6) at 1 × 10^5^ TCID_50_/0.1 mL. The experimental design included four groups: Group 1 (*n* = 4, Apo22-GP-vaccinated, challenged with A/POC/2010); Group 2 (*n* = 2, negative control, challenged with A/POC/2010); Group 3 (*n* = 4, Apo22-GP-vaccinated, challenged with A/GP/2018); and Group 4 (*n* = 2, negative control, challenged with A/GP/2018) (Table 1). Pigs were housed separately and monitored daily for clinical signs of FMD; any animals showing symptoms were isolated immediately. Blood samples were collected at −28, −21, −14, −7, 0, 2, 4, 6, and 8 days post-challenge (dpc). Oral swab samples were collected daily up to 8 dpc using the BD Universal Viral Transport Kit (Becton, Dickinson and Company, Franklin Lakes, NJ, USA). Clinical scores (maximum = 15) were assigned based on the following: body temperature (40 °C: 1 point; >40.5 °C: 2 points; >41 °C: 3 points); lameness (1 point); vesicles on hooves (1–2 points per affected foot); and vesicles on the snout, lips, or tongue (1 point per each affected site).

### 2.9. Evaluation of the Immune Response to the Candidate Vaccine in Pigs

Five pigs aged 8–10 weeks were vaccinated with a dose of the Apo22-GP candidate vaccine (containing 15 μg/mL of antigen). A booster dose was administered at 28 dpv. Sera were collected at 0, 14, 21, 28, 42, 56, and 84 dpv.

### 2.10. Measurement of Structural Protein (SP)-Specific Antibody Levels

Antibodies in serum specific to SPs were measured using the VDPro FMDV A Ab ELISA kit (MEDIAN Diagnostics, Chuncheon, Republic of Korea). According to the criteria provided by the manufacturer, samples with an optical density (OD) value ≥ 0.4 were considered positive for an immune response.

### 2.11. Virus Neutralization Assay

Serum samples were heat-inactivated by exposing them to 56 °C for 30 min. Each sample was incubated with FMDV (100 TCID_50_) for 1 h and then added to LFBK cells. After three days of incubation, the CPE was evaluated. Neutralizing antibody titers were determined by calculating the Log_10_ reciprocal serum dilution required to neutralize a viral load of 100 TCID_50_ [17]. The viruses used in the assay were A/POC/2010, A22 Iraq, A/YC/2017, and A/GP/2018.

### 2.12. Virus Detection in Vaccinated and Challenged Pigs

Real-time PCR was performed on serum and oral swab samples, which were obtained from vaccinated and challenged swine, to determine the presence of FMDV RNA. RNA extraction was conducted using the QIAcube HT instrument (QIAGEN, Hilden, Germany). Quantitative detection was carried out with a one-step prime-script RT-PCR kit (Bioneer, Daejeon, Republic of Korea) in combination with the CFX96 Touch Real-Time PCR Detection System (Bio-Rad, Hercules, CA, USA). For amplification of the 3D region of the FMDV, the forward primer 5′-GGAACYGGGTTTTAYAAACCTGTRAT-3′ and reverse primer 5′-CCTCTCCTTTGCACGCCGTGGGA-3′ were used. The probe sequence was 5′-CCCADCGCAGGTAAAGYGATCTGTA-3′, labeled with 6-FAM at the 5′ end and TAMRA at the 3′ end.

### 2.13. Statistical Analysis

Data analyses were conducted using t-tests and GraphPad Prism (ver. 5.0; GraphPad Software, San Diego, CA, USA) and GraphPad Instant (ver. 3.05; GraphPad Software) software. Statistical analysis was performed using a two-way ANOVA followed by Tukey’s test. Data are presented as the mean ± SEM (standard error of the mean), and significance levels were set at * *p* < 0.05, ** *p* < 0.01, and *** *p* < 0.001.

### 2.14. Ethics Statement

All animal procedures in this study were carried out in strict accordance with the Animal and Plant Quarantine Agency’s (APQA) regulations for the ethical treatment and use of laboratory animals. Experimental protocols received prior approval from the APQA Institutional Animal Care and Use Committee (approval no. 2019-461). Throughout the study, measures were taken to reduce discomfort or distress in the animals to the greatest extent possible.

## 3. Results

### 3.1. Comparison of the Amino Acid Sequences of the Apo22 and Apo22-GP SPs

**Figure 1:**
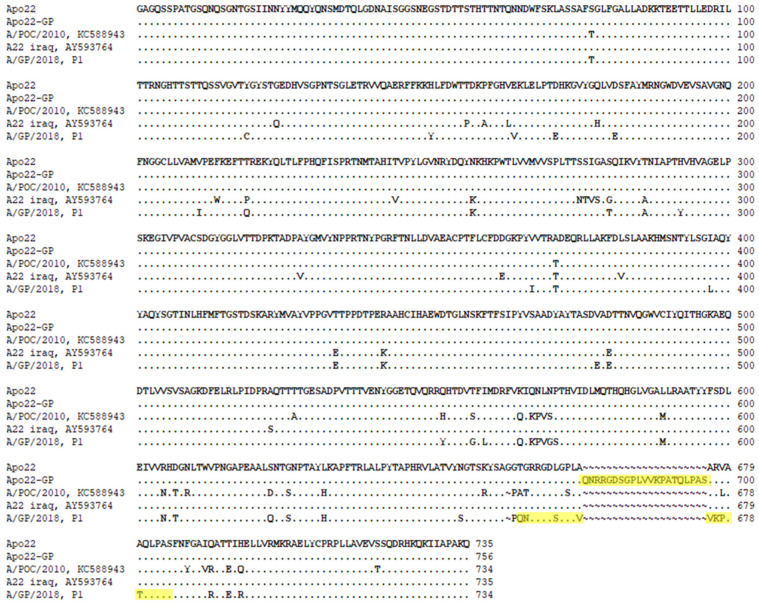
Sequences of the structural proteins (SPs) of the Apo22 and Apo22-GP vaccine strains. The amino acid sequences of the SPs of the vaccine strains are shown alongside those of the incorporated SPs from the A/POC/2010 (VP4, VP2, and VP3) and A22 Iraq (VP1) strains. Yellow box: highlights the amino acid sequence from A/GP/2018 that was inserted into VP1 to create the Apo22-GP strain.

Figure 1 shows the amino acid sequences of the SPs in the Apo22 and Apo22-GP strains. The Apo22-GP strain contained A/POC/2010 VP4, VP2, and VP3, as well as A22 Iraq VP1. Additionally, the RGD site of A/GP/2018 was inserted into the GH loop region of the A22 Iraq VP1 in the Apo22-GP strain.

### 3.2. Development of Chimeric Viruses

**Figure 2:**
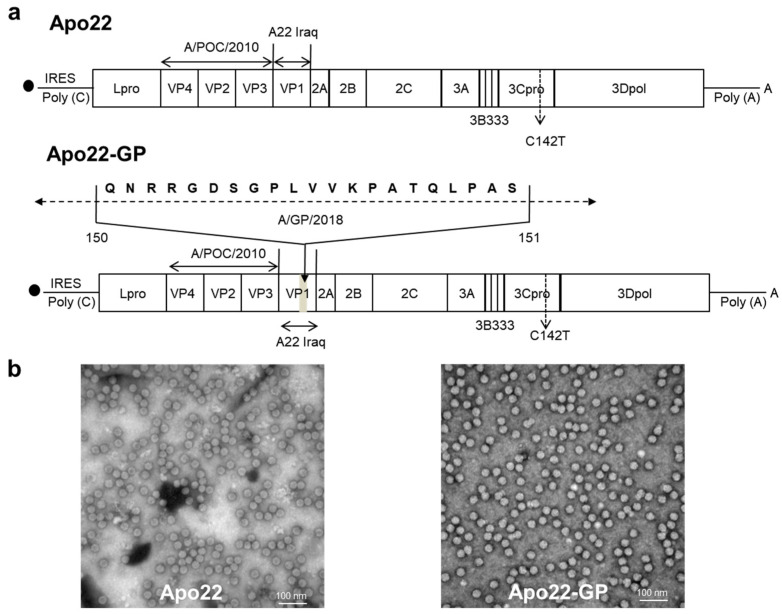
Recombinant Apo22 and Apo22-GP strains as FMD vaccine candidates: (**a**) schematic representation of the structural proteins; (**b**) electron microscopy images of viral particles. Scale bar: 100 nm.

The complete genomic cDNA of the O_1_Manisa strain was cloned into the pBluescript SKII vector, positioned downstream of the T7 RNA polymerase promoter. To construct the Apo22, the capsid protein regions VP4, VP2, and VP3 were replaced with those from A/POC/2010, while VP1 was substituted with that of A22 Iraq. For Apo22-GP, the VP1 segment in Apo22 was further modified by inserting amino acids 140–160 with the equivalent sequence from A/GP/2018 (Figure 2a). In the viral genome, the 3B1B2 segment was replaced with two 3B3 segments, serving as a genetic marker that allowed for differentiation of chimeric viruses from the wild-type viruses using a lateral-flow antigen diagnostic kit designed to detect NSPs. In addition, images captured by electron microscopy revealed that the chimeric viruses had an average diameter of roughly 25 nm. This measurement is consistent with the size observed for the wild-type viruses (Figure 2b).

### 3.3. Protection of Candidate Vaccines in Mice Against Type A Viruses

Mice were immunized with serially diluted doses of Apo22 or Apo22-GP corresponding to 1/10 (1.5 µg of 146 S antigen in 0.1 mL), 1/40, 1/160, 1/640, and, optionally, 1/1280 and 1/2560 of the dose used in pigs. The animals were then challenged with the following four type A FMDV strains: A/POC/2010, A22 Iraq, A/YC/2017, and A/GP/2018. All vaccinated mice survived the challenges with A/POC/2010 and A22 Iraq. Against A/YC/2017, the survival rates of the Apo22-vaccinated mice were 75%, 50%, and 0% at 1/640, 1/1280, and 1/2560 doses, whereas all Apo22-GP-vaccinated mice survived. The PD_50_ values for Apo22 were >128.0 against A/POC/2010, and for A22 Iraq, 107.6 against A/YC/2017, and 238.9 against A/GP/2018; for Apo22-GP, they were >128.0 and >362.0, respectively (Table 2). The low-dose Apo22 group (1/1280 and 1/2560) exhibited weight loss after A/YC/2017 challenge, while the Apo22-GP-vaccinated mice maintained a stable weight following all challenges (Supplementary Figures S1 and S2). Collectively, Apo22-Gp provided stronger protection, particularly against A/YC/2017 and A/GP/2018, than Apo22.

### 3.4. Protective Efficacy of the Apo22-GP Vaccine Against Type A FMDV Challenge in Pigs

**Figure 3:**
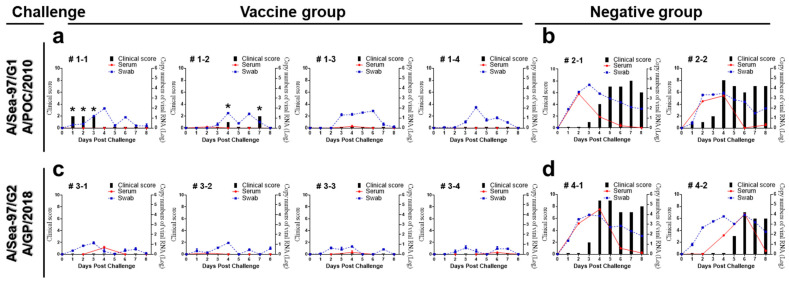
Clinical sign scores, viremia levels, and oral virus shedding in vaccinated and control pigs challenged with A/POC/2010 or A/GP/2018: (**a**) vaccinated pigs (*n* = 4) challenged with the A/POC/2010 virus; (**b**) unvaccinated controls (*n* = 2) challenged with the A/POC/2010 virus; (**c**) vaccinated pigs (*n* = 4) challenged with the A/GP/2018 virus; (**d**) negative controls (*n* = 2) challenged with the A/GP/2018 virus. * Temporary body temperature increase. Black bars: clinical signs; blue: oral swab; red: serum samples.

Four weeks after vaccination with the Apo22-GP candidate vaccine, pigs were subjected to a challenge with type A viruses (Table 2). Clinical symptom scores in the unvaccinated group were elevated compared to the vaccinated group, from 3 to 8 dpc. Specifically, a transient increase in body temperature was noted in two of the four pigs challenged with the A/POC/2010 virus. All vaccinated pigs displayed virus shedding. Quantitative analyses of the viral RNA shedding showed that the total viral RNA load (AUC) was markedly lower in vaccinated pigs than in unvaccinated controls for both challenge viruses (Supplementary Figure S3). Furthermore, unvaccinated pigs showed persistently mild clinical signs at the injection site from 3 dpc through to the end of the 8-day observation period (Figure 3).

### 3.5. Neutralizing Antibody Response to Apo22-GP Vaccination and Type A Challenge in Pigs

**Figure 4:**
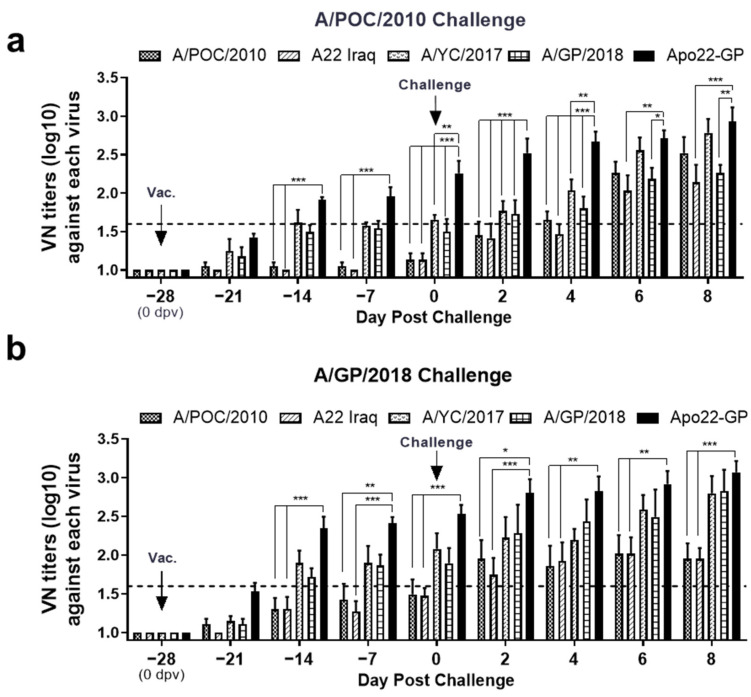
Virus-neutralizing-antibody response to wild-type FMDV challenge in Apo22-GP-vaccinated pigs. Sera were collected at multiple points dpc from pigs immunized with Apo22-GP and challenged with either (**a**) A/POC/2010 (n = 4) or (**b**) A/GP/2018 (n = 4). Neutralizing-antibody levels were measured against five type A FMDV strains. Titers are expressed as Log_10_ values; values ≥ 1.6 Log_10_ (dotted line) were considered positive. Statistical analysis was performed using two-way ANOVA followed by Tukey’s test. * *p* < 0.05; ** *p* < 0.01; *** *p* < 0.001.

Four weeks after inoculation with the Apo22-GP vaccine, the levels of antibodies that neutralized four different viruses had increased. Notably, higher levels of antibodies specific to the A/ASIA/Sea-97 G2 sublineage viruses were observed. Following challenge with the A/POC/2010 (A/ASIA/Sea-97 G1) virus, the titers of antibodies that could effectively neutralize both the G1 and G2 sublineage viruses increased, showing that a consistent response across both sublineages was induced. The levels of antibodies that could neutralize the Apo22-GP virus were similar in all pigs (titer > 1.6 Log_10_), as shown in Figure 4. Control data are provided in Supplementary Figure S4.

### 3.6. Assessment of Neutralizing Antibodies Levels Induced by the Apo22-GP Vaccine in Pigs

**Figure 5:**
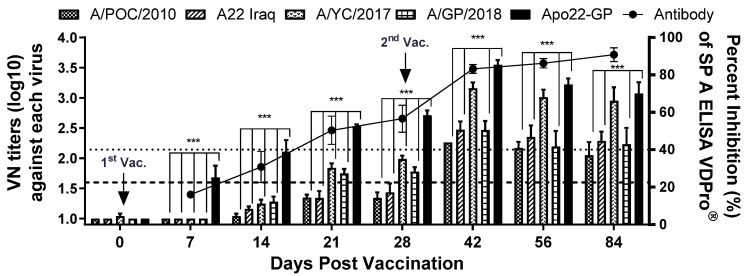
Immunogenicity of the Apo22-GP vaccine in pigs. Serological responses were assessed in pigs (*n* = 5) vaccinated with the Apo22-GP candidate. Neutralizing-antibody levels against various type A FMDVs and levels of SP-specific antibodies were quantified. Titers ≥ 1.6 Log_10_ (dotted line) were considered positive for virus neutralization. SP-specific antibody levels were measured by ELISA, with a threshold of 40% defined as positive. Statistical analysis was performed using two-way ANOVA followed by Tukey’s test. *** *p* < 0.001.

The levels of antibodies specific for SPs and virus-neutralizing antibodies in pigs vaccinated with the Apo22-GP candidate vaccine were quantitatively compared. The results are shown in Figure 5. After the first dose, the levels of neutralizing antibodies effective against the A/ASIA/Sea-97 G1 and G2 sublineage viruses varied. However, after the second dose, the levels of neutralizing antibodies effective against the G1 and G2 sublineage viruses showed similar trends. A marked increase in the antibodies capable of neutralizing the A/YC/2017 virus was observed. After the second dose, the neutralizing-antibody titer exceeded 2.0 Log_10_ for all four tested viruses.

## 4. Discussion

In the pool 1 region, which includes East Asia, the predominant FMDV serotypes are O, A, and Asia1 [5,6]. Frequent livestock movement and active trade among countries in this region facilitate rapid virus transmission [18]. According to the World Reference Laboratory for Foot-and-Mouth Disease (WRLFMD), FMD viruses identified as belonging to the A/ASIA/Sea-97 lineage have been reported in the pool 1 region, including Cambodia (2007, 2009, 2015, and 2017), China (2009, 2013, 2014, and 2018), the Republic of Korea (2010, 2017, and 2018), and Thailand (2005–2023) [19]. Viruses from the A/ASIA/Sea-97 G1 sublineage were first identified in Thailand and later detected in Malaysia and the Republic of Korea [12,13], while those from the A/ASIA/Sea-97 G2 sublineage were reported in China (2013), Russia (2014), Vietnam (2016), and the Republic of Korea (2017 and 2018) [11]. Recent reports indicate that the A/ASIA/Sea-97 lineage is dominant in the pool 1 region, accounting for approximately 18% of infections from 2024 to 2025 [20].

The A/YC/2017 strain, circulating in the Republic of Korea, showed antigenic mismatch with several commercial vaccine strains, such as the 2D-VNT A22, A Malaysia 97, A TUR 06, and A IRAN 05 strains [11]. While the A/YC/2017 strain mainly infects cattle, the A/GP/2018 strain affects pigs; nevertheless, their VP1 sequences share ~95.7% similarity. Thus, the A/GP/2018 strain has emerged as a promising vaccine candidate in Asia due to its strong protective potential.

In this study, we developed a chimeric candidate designed to protect against both the G1 and G2 sublineages of the A/ASIA/Sea-97 lineage. To enhance efficacy against the predominant G2 viruses, the VP1 region of the A/GP/2018 strain, including its RGD site, was inserted into the previously developed Apo22 strain. In mice, Apo22-GP demonstrated superior protection against G2 sublineage viruses compared with Apo22. In pigs, a single dose provided effective clinical protection against both the A/POC/2010 and A/GP/2018 viruses, despite transient virus shedding, as vaccinated pigs showed no vesicular lesions and only short-term fever, whereas control pigs developed typical FMD symptoms.

Although cellular immune responses were not evaluated in this study, previous reports have shown that Th1-type cytokines, including interferon-γ, tumor necrosis factor-α, interleukin-12, and interleukin-15, are associated with protection against FMDV [21,22,23]. Two doses of the vaccine resulted in neutralizing-antibody titers exceeding 2.0 Log10 for both G1 and G2 sublineage viruses, indicating protective potential against the type A FMDV strains circulating in Asia.

Because viral mutations can reduce the effectiveness of existing vaccines, recombinant vaccine development is essential for regionally tailored FMD control strategies [24]. Extensive efforts are underway to produce vaccines that provide broad and durable protection against FMD [25,26,27]. The present study contributes to this effort by introducing a novel recombinant vaccine candidate specifically designed for the A/ASIA/Sea-97 lineage. Based on these findings, the Apo22-GP candidate vaccine appears to be superior to the previously reported Apo22-B [14] vaccine and represents a promising option for FMD control in East Asia.

Our results suggest that further experiments are needed to evaluate the protection conferred by the vaccine using a broader range of A/ASIA/Sea-97 viruses circulating in Asia. Given the limited number of animals used and the observation of transient virus shedding, additional studies with larger sample sizes and diverse challenge strains are needed to strengthen the generalizability of the findings. Furthermore, the transient detection of viral RNA in vaccinated pigs indicates that sterilizing immunity was not achieved, although the total viral load was markedly lower than that in unvaccinated controls. In addition, these studies should investigate the underlying immune mechanisms related to protection. Furthermore, future studies should include a commercial vaccine group for direct comparison to validate the relative efficacy of the candidate vaccine. Moreover, as the present study focused on pigs, future studies in other livestock species, such as cattle and goats, are necessary. Finally, it is necessary to investigate the optimization of the administration route to reduce pathological lesions [13].

## 5. Conclusions

In pig challenge experiments conducted in pigs, a newly developed FMD vaccine candidate, Apo22-GP, provided protection against viruses from the A/ASIA/Sea-97 G1 and G2 sublineages circulating in Asia. In addition, immunogenicity studies confirmed that the vaccine elicited a strong neutralizing-antibody response against A/ASIA/Sea-97. Therefore, this study provides an important contribution to the development of regionally customized FMD vaccines and offers a promising strategy for the effective control of A/ASIA/Sea-97 viruses prevalent in East Asia.

## Figures and Tables

**Table 1:** Experimental designs for the evaluation of the Apo22-GP vaccine’s efficacy.

Group (Cage)	No. of Animals	Experimental Group	Challenge Virus (1 × 10^5^ TCID_50_/0.1 mL)	Days of Blood Collection (dpc)	Days of Oral Swab Collection (dpc)	Comments
1 (1)	4	Apo22-GP	A/POC/2010	−28, −21, −14, −7, 0, 2, 4, 6, 8	0~8	Animals exhibiting clinical signs after challenge were isolated in an empty cage.
2 (1)	2	Negative group				
3 (2)	4	Apo22-GP	A/GP/2018			
4 (2)	2	Negative group				

**Table 2:** Determination of 50% protective dose (PD_50_) of the vaccine candidate in immunized mice.

Vaccine Candidate	Challenge Viruses (PD_50_)			
	G1 (A/POC/2010) ^(1)^	A22 IRAQ ^(1)^	G2 (A/YC/2017) ^(2)^	G2 (A/GP/2018) ^(2)^
Apo22	>128.0 *	>128.0 *	107.6	238.9
Apo22-GP	>128.0 *	>128.0 *	>362.0	>362.0

The candidate vaccines Apo22 and Apo22-GP were administered to groups of C57BL/6 mice (*n* = 4 or 5 per group) via intramuscular injection, using doses of 1/10, 1/40, 1/160, and 1/640 (optional: 1/1280 and 1/2560). A challenge was subsequently performed using the following four type A FMDVs: A/POC/2010, A22 Iraq, A/YC/2017, and A/GP/2018 (1 × 10^5^ TCID_50_/0.1 mL). The survival rates and body weights of the animals were recorded for a period of seven days post-challenge (data are shown in Supplementary Figures S1 and S2). * The animal experiment was conducted at a dose of up to 1/640 doses. ^(1)^ In the case of the first experiment, all cases were protected. ^(2)^ Additional animal experiments were conducted at 1/1280 and 1/2560 doses.

## Data Availability

Data are contained within the article.

## Supplementary Materials

Additional supplementary data can be accessed at the following link: https://www.mdpi.com/article/10.3390/vaccines13111104/s1, Table S1: Number of mice used in each dose group for PD_50_ experiments; Figure S1: Estimation of the PD_50_ in mice immunization with the Apo22 vaccine and subsequently challenged with type A FMDVs; Figure S2: Estimation of the PD_50_ in mice immunization with the Apo22-GP vaccine and subsequently challenged with type A FMDVs; Figure S3: Viral RNA copy numbers in serum and oral swabs from vaccinated (*n* = 4) and unvaccinated (*n* = 2) pigs after challenge with type A viruses; Figure S4: Virus-neutralizing-antibody levels in pigs challenged with wild-type FMDV. Sera were collected at multiple points dpc from pigs challenged with either (a) A/POC/2010 (*n* = 2) or (b) A/GP/2018 (*n* = 2).

## References

[1] Abubakar M., Syed Z., Manzoor S., Arshed M.J. (2022). Deciphering Molecular Dynamics of Foot and Mouth Disease Virus (FMDV): A Looming Threat to Pakistan’s Dairy Industry. Dairy.

[2] Jamal S.M., Belsham G.J. (2013). Foot-and-mouth disease: Past, present and future. Vet. Res..

[3] Paton D.J., Valarcher J.F., Bergmann I., Matlho O.G., Zakharov V.M., Palma E.L., Thomson G.R. (2005). Selection of foot and mouth disease vaccine strains—A review. Rev. Sci. Tech..

[4] Paton D.J., Sumption K.J., Charleston B. (2009). Options for control of foot-and-mouth disease: Knowledge, capability and policy. Philos. Trans. R. Soc. Lond. B Biol. Sci..

[5] Silas Lendzele S. (2022). Foot and Mouth Disease in Cameroon: A Systematic Review to Support its Progressive Control. J. Clin. Vet. Res..

[6] Hammond J.M., Maulidi B., Henning N. (2021). Targeted FMD Vaccines for Eastern Africa: The AgResults Foot and Mouth Disease Vaccine Challenge Project. Viruses.

[7] Lyons N.A., Ludi A.B., Wilsden G., Hamblin P., Qasim I.A., Gubbins S., King D.P. (2017). Evaluation of a polyvalent foot-and-mouth disease virus vaccine containing a Saudi-95 against field challenge on large-scale dairy farms in Saudi Arabia with the emerging A/ASIA/G-VII viral lineage. Vaccine.

[8] Mahapatra M., Parida S. (2018). Foot and mouth disease vaccine strain selection: Current approaches and future perspectives. Expert Rev. Vaccines.

[9] Fernandez-Sainz I., Gavitt T.D., Koster M., Ramirez-Medina E., Rodriguez Y.Y., Wu P., Silbart L.K., de Los Santos T., Szczepanek S.M. (2019). The VP1 G-H loop hypervariable epitope contributes to protective immunity against Foot and Mouth Disease Virus in swine. Vaccine.

[10] Mahapatra M., Statham B., Li Y., Hammond J., Paton D., Parida S. (2016). Emergence of antigenic variants within serotype A FMDV in the Middle East with antigenically critical amino acid substitutions. Vaccine.

[11] Hwang J.H., Lee G., Kim A., Park J.H., Lee M.J., Kim B., Kim S.M. (2021). A Vaccine Strain of the A/ASIA/Sea-97 Lineage of Foot-and-Mouth Disease Virus with a Single Amino Acid Substitution in the P1 Region That Is Adapted to Suspension Culture Provides High Immunogenicity. Vaccines.

[12] Bae S., Li V., Hong J., Kim J.N., Kim H. (2021). Phylogenetic and evolutionary analysis of foot-and-mouth disease virus A/ASIA/Sea-97 lineage. Virus Genes.

[13] Kim D.W., Cho G., Kim H., Lee G., Lim T.G., Kwak H.Y., Park J.H., Park S.H. (2023). Immunogenicity and Protection against Foot-and-Mouth Disease Virus in Swine Intradermally Vaccinated with a Bivalent Vaccine of Foot-and-Mouth Disease Virus Type O and A. Vaccines.

[14] Shin S.H., Hwang S.Y., Kim H.M., Shin S.H., Ko M.K., Lee M.J., Kim S.M., Park J.H. (2024). Evaluation of a Vaccine Candidate Designed for Broad-Spectrum Protection against Type A Foot-and-Mouth Disease in Asia. Vaccines.

[15] Lee S.Y., Lee Y.J., Kim R.H., Park J.N., Park M.E., Ko M.K., Choi J.H., Chu J.Q., Lee K.N., Kim S.M. (2017). Rapid Engineering of Foot-and-Mouth Disease Vaccine and Challenge Viruses. J. Virol..

[16] Ito N., Takayama-Ito M., Yamada K., Hosokawa J., Sugiyama M., Minamoto N. (2003). Improved recovery of rabies virus from cloned cDNA using a vaccinia virus-free reverse genetics system. Microbiol. Immunol..

[17] WOAH Manual of Diagnostic Tests and Vaccines for Terrestrial Animals, Chapter 3.1.8 Foot and Mouth Disease. https://www.woah.org/fileadmin/Home/eng/Health_standards/tahm/3.01.08_FMD.pdf.

[18] Aslam M., Alkheraije K.A. (2023). The prevalence of foot-and-mouth disease in Asia. Front. Vet. Sci..

[19] WRLFMD Country Reports. https://www.wrlfmd.org/country-reports/east-and-southeast-asia.

[20] WRLFMD Quarterly Reports. https://www.wrlfmd.org/2025-quarter-2-apr-jun.

[21] Parida S., Oh Y., Reid S.M., Cox S.J., Statham R.J., Mahapatra M., Anderson J., Barnett P.V., Charleston B., Paton D.J. (2006). Interferon-gamma production in vitro from whole blood of foot-and-mouth disease virus (FMDV) vaccinated and infected cattle after incubation with inactivated FMDV. Vaccine.

[22] Oh Y., Fleming L., Statham B., Hamblin P., Barnett P., Paton D.J., Park J.H., Joo Y.S., Parida S. (2012). Interferon-γ induced by in vitro re-stimulation of CD4+ T-cells correlates with in vivo FMD vaccine induced protection of cattle against disease and persistent infection. PLoS ONE.

[23] Zhang L., Feng X., Jin Y., Ma J., Cai H., Zhang X. (2016). Immunoprotective mechanisms in swine within the “grey zone” in antibody response after immunization with foot-and-mouth disease vaccine. Virus Res..

[24] Saeed A., Kanwal S., Arshad M., Ali M., Shaikh R.S., Abubakar M. (2015). Foot-and-mouth disease: Overview of motives of disease spread and efficacy of available vaccines. J. Anim. Sci. Technol..

[25] Childs K., Harvey Y., Waters R., Woma T., Wilsden G., Sun H., Sun P., Seago J. (2023). Development of a quadrivalent foot-and-mouth disease vaccine candidate for use in East Africa. Vaccine.

[26] Shao J., Liu W., Gao S., Chang H., Guo H. (2024). A recombinant multi-epitope trivalent vaccine for foot-and-mouth disease virus serotype O in pigs. Virology.

[27] Zha J., Zhang K., Zhang X., Sun P., Li D., Cao Y., Bai X., Ma X., Li K., Yuan H. (2025). Foot-and-mouth disease virus vaccine with VP1 G-H loop substitution of the Cathay strain broadens antigen spectrum. Appl. Microbiol. Biotechnol..

