# Supplementary material for: Development of a Chimeric Vaccine Providing Protection Against the Type A ASIA/Sea-97 FMDVs in East Asia
Source: Vaccines (Basel). 2025 Oct 29;13(11):1104. doi: 10.3390/vaccines13111104 (PMC12656955; doi:10.3390/vaccines13111104)
Supplement: Supplementary file 1 [file vaccines-13-01104-s001.zip › vaccines-3825817-supplementary.pdf]

Supplementary Table S1. Number of mice used in each dose group for PD_{50} experiments

Vaccine candidates	Challenge viruses	Dose group	No. of mice*
Apo22	G1 (A/POC/2010)	1/40, 1/160, 1/640	4, 4, 4
		Negative Control (NC)	4
	A22 Iraq	1/40, 1/160, 1/640	4, 4, 4
		Negative Control (NC)	3
	G2 (A/YC/2017)	1/40, 1/160, 1/640, 1/1280, 1/2560	4, 4, 4, 4, 4
		Negative Control (NC)	4
	G2 (A/GP/2018)	1/10, 1/40, 1/160, 1/640, 1/1280, 1/2560	5, 5, 5, 5, 5, 5
		Negative Control (NC)	5
Apo22-GP	G1 (A/POC/2010)	1/10, 1/40, 1/160, 1/640	5, 5, 5, 5
		Negative Control (NC)	4
	A22 Iraq	1/40, 1/160, 1/640	4, 4, 4
		Negative Control (NC)	5
	G2 (A/YC/2017)	1/40, 1/160, 1/640, 1/1280, 1/2560	4, 4, 4, 4, 4
		Negative Control (NC)	4
	G2 (A/GP/2018)	1/10, 1/40, 1/160, 1/640, 1/1280, 1/2560	5, 5, 5, 5, 5, 5
		Negative Control (NC)	4

Supplementary S1 : Estimation of the PD_{50} in mice immunization with the Apo22 vaccine and subsequently challenged with type A FMDVs.
*Because of the vendor's breeding schedule and limitations in littler size, it was not feasible to initiated all experiments simultaneously; therefore, animal were used sequentially. Accordingly, the group sizes were set at 3-5 mice.

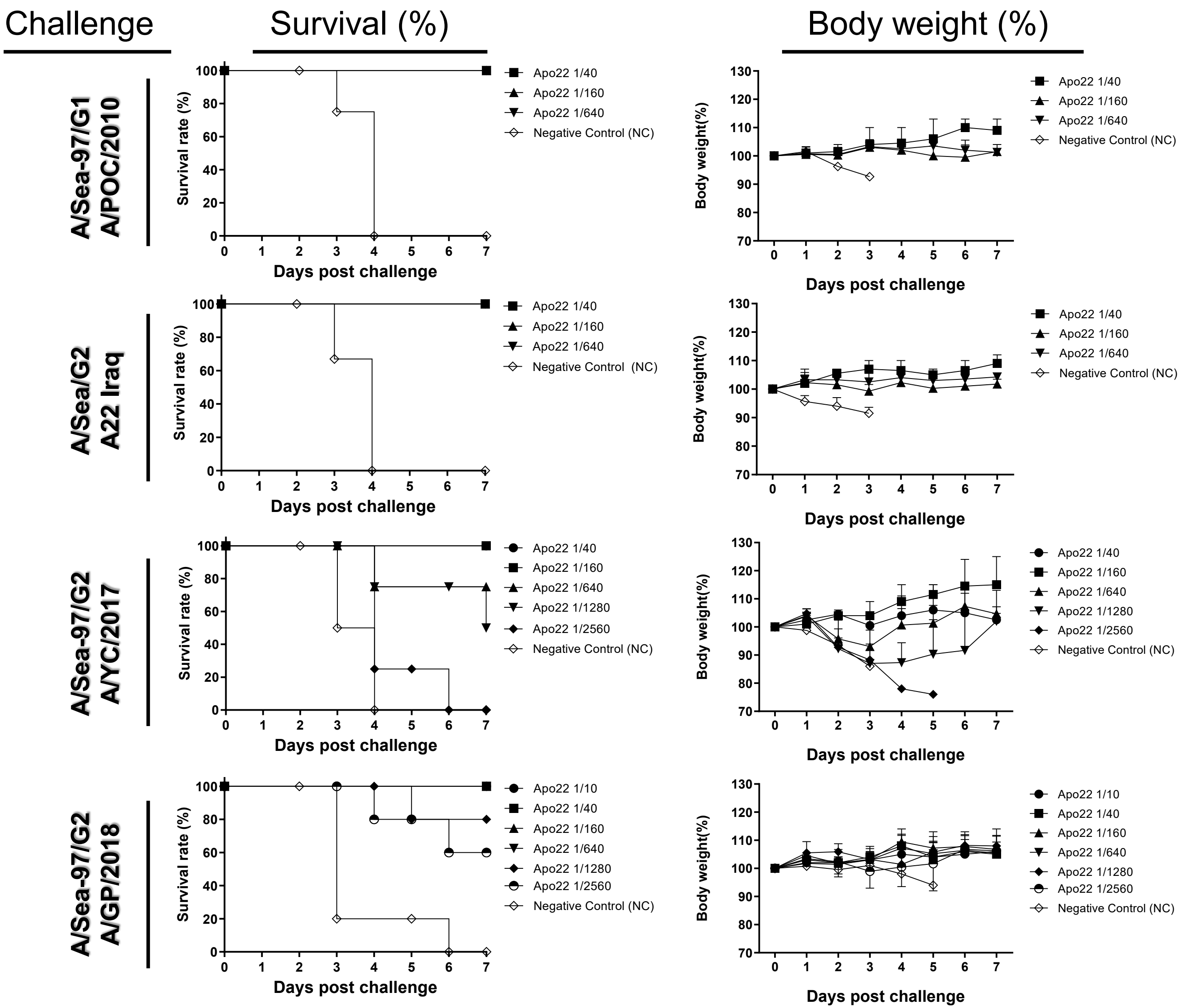

Supplementary Figure S1 : Estimation of the PD_{50} in mice immunization with the Apo22 vaccine and subsequently challenged with type A FMDVs. The survival rate and change of body weight challenge with 1×10^5 $TCID_{50}$/0.1 mL of A/POC/2010, A22 Iraq, A/YC/2017 and A/GP/2018.

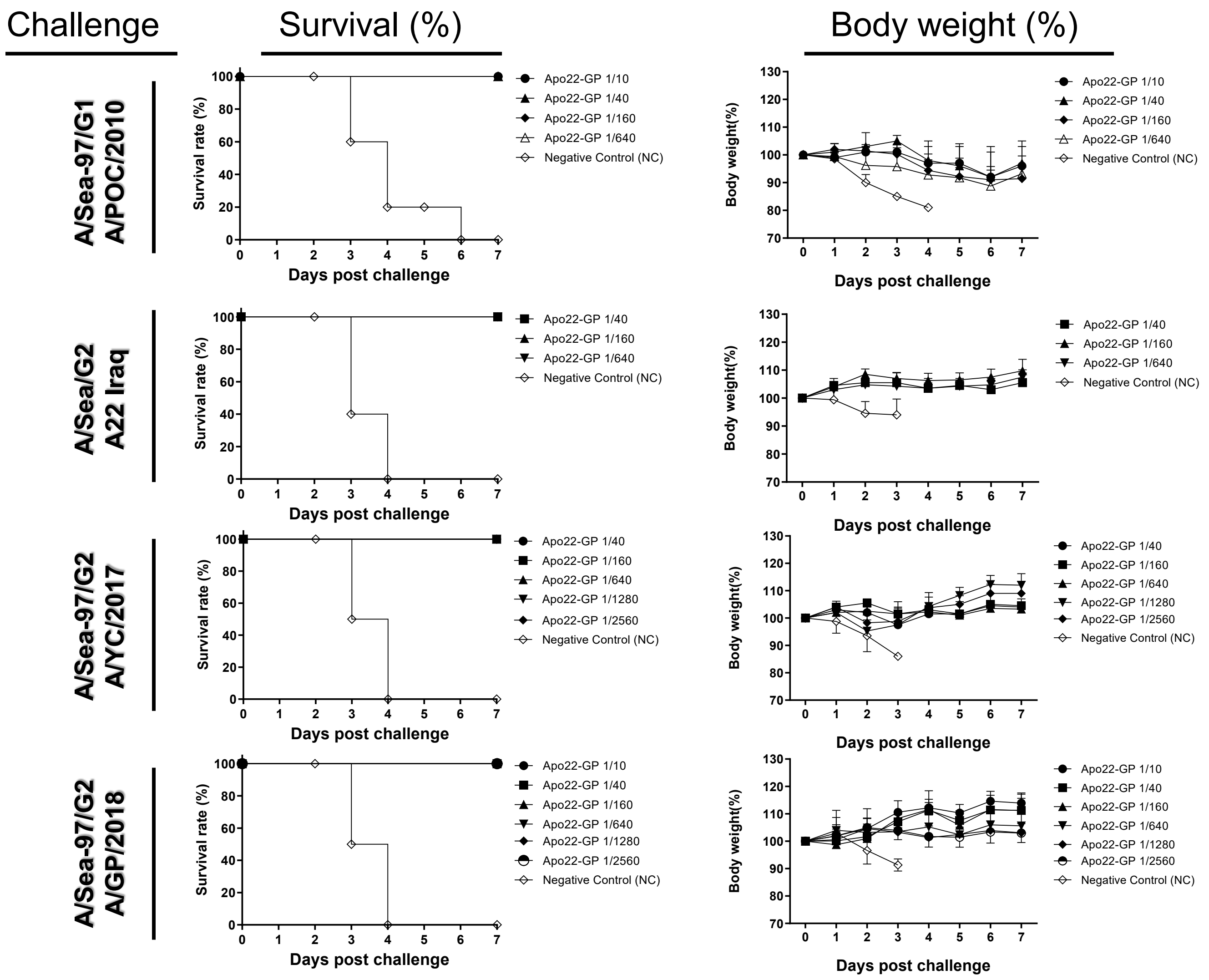

Supplementary Figure S2 : Estimation of the PD_{50} in mice immunization with the Apo22-GP vaccine and subsequently challenged with type A FMDVs The survival rate and change of body weight challenge with 1×10^5 $TCID_{50}$/0.1 mL of A/POC/2010, A22 Iraq, A/YC/2017 and A/GP/2018.

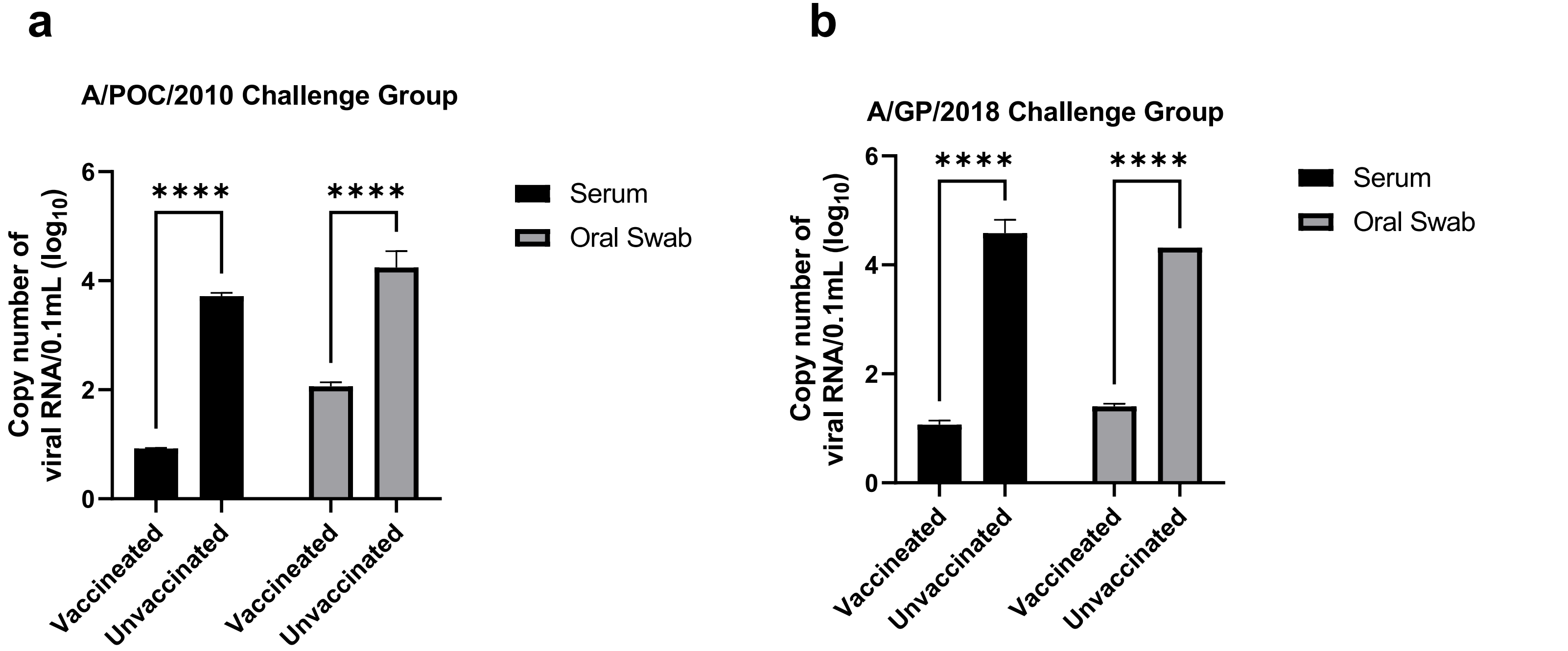

Supplementary Figure S3 : Viral RNA copy numbers in serum and oral swabs from vaccinated (n = 4) and unvaccinated (n = 2) pigs after challenge with type A viruses.

(a) A/POC/2010 challenge group; (b) A/GP/2018 challenge group. Viral RNA level were quantified by qPT-PCR and expressed as log_{10} copies per 0.1 mL. Statistical analysis was performed using two-way ANOVA followed by Turkey's test. ****p<0 .0 0 0 1.

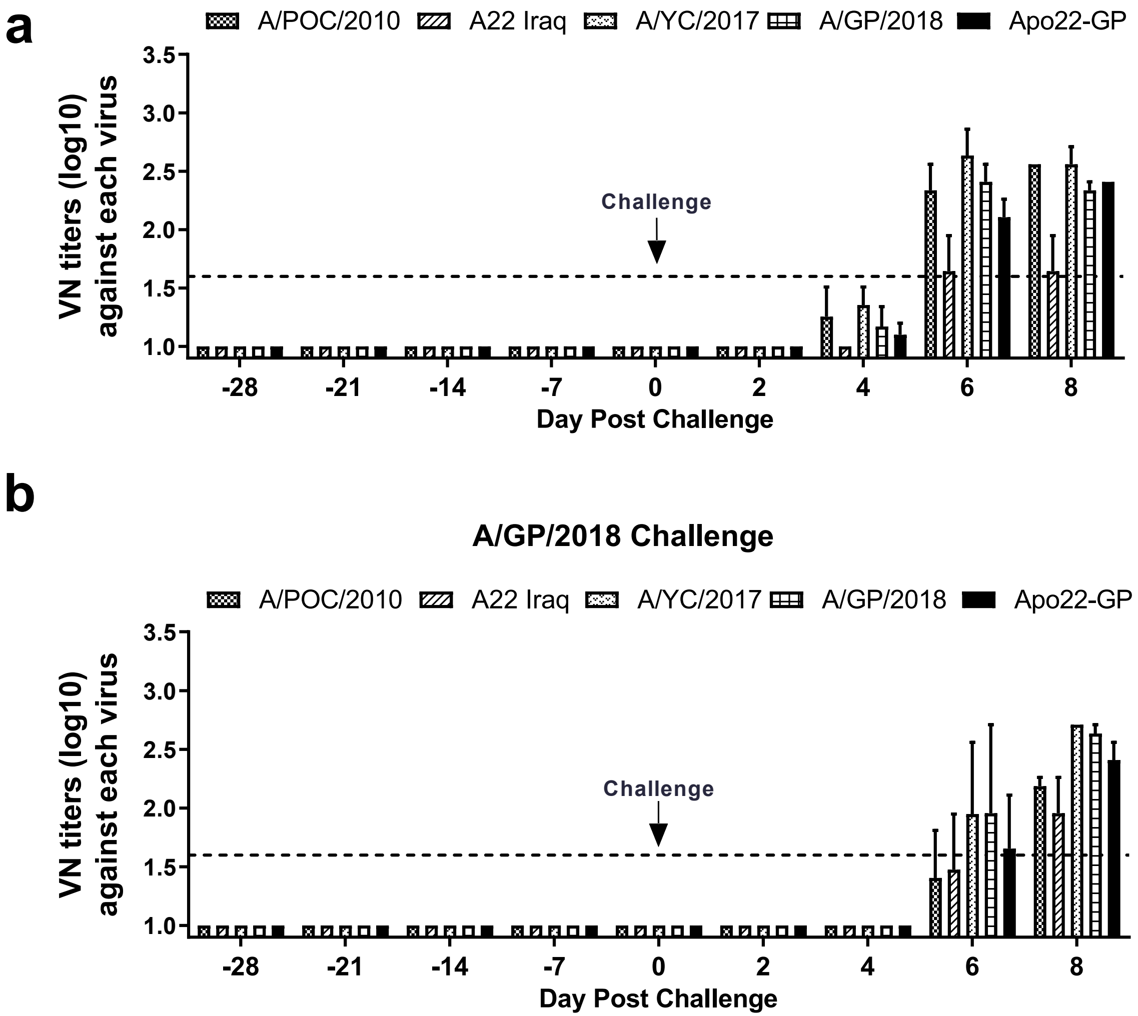

Supplementary Figure S4 : Virus neutralizing antibody levels in pigs challenged with wild type FMDV. Sera were collected at multiple points dpc from pigs challenged with either A/POC/2010 (a; n = 2) or A/GP/2018 (b; n = 2). Neutralizing antibody levels were measured against five type A FMDV strains: A/POC/2010, A22 Iraq, A/YC/2017, A/GP/2018, and Apo22-GP. Titers are expressed as $\log_{10}$ values; values ≥ 1.6 $\log_{10}$ (dotted line) were considered positive.
